# Supplementary material for: Bovine Enteroids as an In Vitro Model for Infection with Bovine Coronavirus
Source: Viruses. 2023 Feb 27;15(3):635. doi: 10.3390/v15030635 (PMC10054012; doi:10.3390/v15030635)
Supplement: Supplementary file 1 [file viruses-15-00635-s001.zip › viruses-2201567-supplementary.pdf]

**Table S1.** Formulations of media that were tested for growth (medium 1) and differentiation (media 1–4).

| Medium | Growth Factors/Additives (Concentration) | Producer | Reference |
|---|---|---|---|
| 1. IntestiCult Organoid Growth Medium (Mouse) | | STEMCELL Technologies, Cambridge, UK | |
| | Gentamicin (50 µg/mL) | Life Technologies, Paisley, Scotland, UK | |
| | ROCK inhibitor Y-27632 (10 µM) | Cayman chemicals, Ann arbor, MI, USA | [18] |
| | A83-01/LY2157299 (0.5 µM) | Cayman chemicals | |
| | SB202190 (10 µM) | Enzo Life Science, England, UK | |
| 2. IntestiCult Organoid Growth Medium (Mouse) | | STEMCELL Technologies | [17] |
| | Human recombinant R-spondin (1 µg/mL) | R&D systems, Minneapolis, MIN, USA | |
| | Murine noggin (100 ng/mL) | PeproTech, London, UK | |
| | Murine epidermal growth factor (EGF) (100 ng/mL) | Merck, Darmstadt, Germany | |
| | CHIR99021 (1.5 µM) | Sigma-Aldrich, St. Louis, MO, USA | |
| | ROCK inhibitor Y-27632 (5 µM) | Cayman chemicals | |
| | SB202190 (5 µM) | Enzo Life Science | |
| | A83-01/LY2157299 (250 nm) | Cayman chemicals | |
| | Primocin (100 µg/mL) | Invivogen, Toulouse, France | |
| 3. Advanced DMEM F12 | | Thermo Fisher Scientific | [21] |
| | FBS (20%) | Thermo Fisher Scientific | |
| | GlutaMAX (2 mM) | Thermo Fisher Scientific | |
| | Pen/str (100 U/mL, 1%) | Life Technologies | |
| | ROCK inhibitor Y-27632 (10 µM) | Cayman chemicals | |
| 4. Advanced DMEM F12 | | Thermo Fisher Scientific | [34] |
| | Pen/str (100 U/mL) | Life Technologies | |
| | HEPES buffer (10 mM) | Life Technologies | |
| | GlutaMAX (1x) | Thermo Fisher Scientific | |
| | Murine epidermal growth factor (EGF) (50 ng/mL) | Merck | |
| | Murine noggin (50 ng/mL) | PeproTech | |
| | Human recombinant R-spondin (200 µg/mL) | R&D systems | |
| | A-83-01/LY2157299 (500 nM) | Cayman chemicals | |
| | B27 supplement (50x), minus Vitamin A (1X) | Thermo Fisher Scientific | |
| | Gastrin I (10 nm) | Sigma-Aldrich | |
| | N2 supplement (1X) | R&D systems | |
| | N-acetylcysteine (1 mM) | R&D systems | |

* Conditioned medium was prepared using L-WRN cells (ATCC CRL-3276) that secrete Wnt3-a, R-spondin 3 and Noggin [35].

**Table S2.** Nucleotide sequences of the primers and probe, and RT-qPCR cycling conditions used for the amplification of BCoV RNA.

| Primers/probe (final concentration) | RT-qPCR cycling conditions | Sequences 5′ to 3′ | Reference |
|---|---|---|---|
| BCoV1F20 (400 nM) | 50 °C for 30 min, 95 °C for 2 min, 40 cycles of 95 °C for 15 s, 55 °C for 30 s, 60 °C for 15 s | TGGTGTCTATATTCATTTCTGCTG | [38] |
| BCoV1R89 (400 nM) | | GGCCACTGCCTAGGATACA | |
| BCoV1P48 (800 nM) | | {FAM}ACACGTCCCTGGCTGAAAGCTG{BHQ1} | |

*FAM: 6-carboxyfluorescein; BHQ: Black hole quencher.

**Table S3.** Primers used for qPCR in the expression study of targeted *Bos taurus* genes.

| Gene type | Gene name | Gene symbol | Ensembl database ID/Refseq ID | Primer sequence (5′-3′) | Reference |
|---|---|---|---|---|---|
| House-keeping genes | 18S ribosomal RNA | 18S rRNA | NR_036642 | Fw: AAACGGCTACCACATCCAAG<br>Rv: CCTCCAATGGATCCTCGTTA | [31] |
| | Glyceraldehyde 3-phosphate dehydrogenase | GAPDH | ENSBTAG00000014731 | Fw: ATCTCGCTCCTGGAAGATG<br>Rv: TCGGAGTGAACGGATTCG | [32] |
| | Beta-actin | ACTB | ESBTAG00000026199 | Fw: ACACCGCAACCAGTTCGCCAT<br>Rv: GTCAGGATGCCTCTCTTGCT | [33] |
| Differentiation markers | Leucine-rich repeat-containing G protein-coupled receptor 5 | LGR5 | ENSBTAG00000013256 | Fw: GTGTTCAGAGCCGCAGTGTA<br>Rv: GATTCCGAAGCAAAAATGGA | [31] |
| | Chromogranin A | ChrA | ENSBTAG00000009836 | Fw: TCTCAATCCTGCGACATCAG<br>Rv: CTGTCTCCGTCCGAGTCTTC | [31] |
| | Mucin 2 | MUC2 | ENSBTAG00000050212 | Fw: TTCGACGGGAGGAAGTACAC<br>Rv: TTCACCGTCTGCTCATTCAG | [31] |
| Immune genes | Interleukin 8 | IL8 | ENSBTAG00000019716 | Fw: CCACACCTTTCCACCCCAAA<br>Rv: CTTGCTTCTCAGCTCTCTTC | [40] |
| | Interleukin 1, alpha | IL1A | ENSBTAG00000010349 | Fw: CTCTCTCAATCAGAAGTCCTTCTATG<br>Rv: CATGTCAAATTTCACTGCCTCCTCC | [41] |
| | Matrix Metallopeptidase 13 | MMP13 | ENSBTAG00000015059 | Fw: GGAACTAAAGAGCACGGTGAC<br>Rv: GGCAGCGACAAGAAACAAG | [33] |
| | Chemokine (C-X-C motif) ligand 3 | CXCL3 | ENSBTAG00000037778 | Fw: GCC ATT GCC TGC AAA CTT<br>Rv: TGC TGC CCT TGT TTA GCA | [42] |
| | Tumor necrosis factor | TNF- $\alpha$ | ENSBTAG00000025471 | Fw: CCAGAGGGAAGAGCAGTCC<br>Rv: GGAGAGTTGATGTCGGCTAC | [43] |

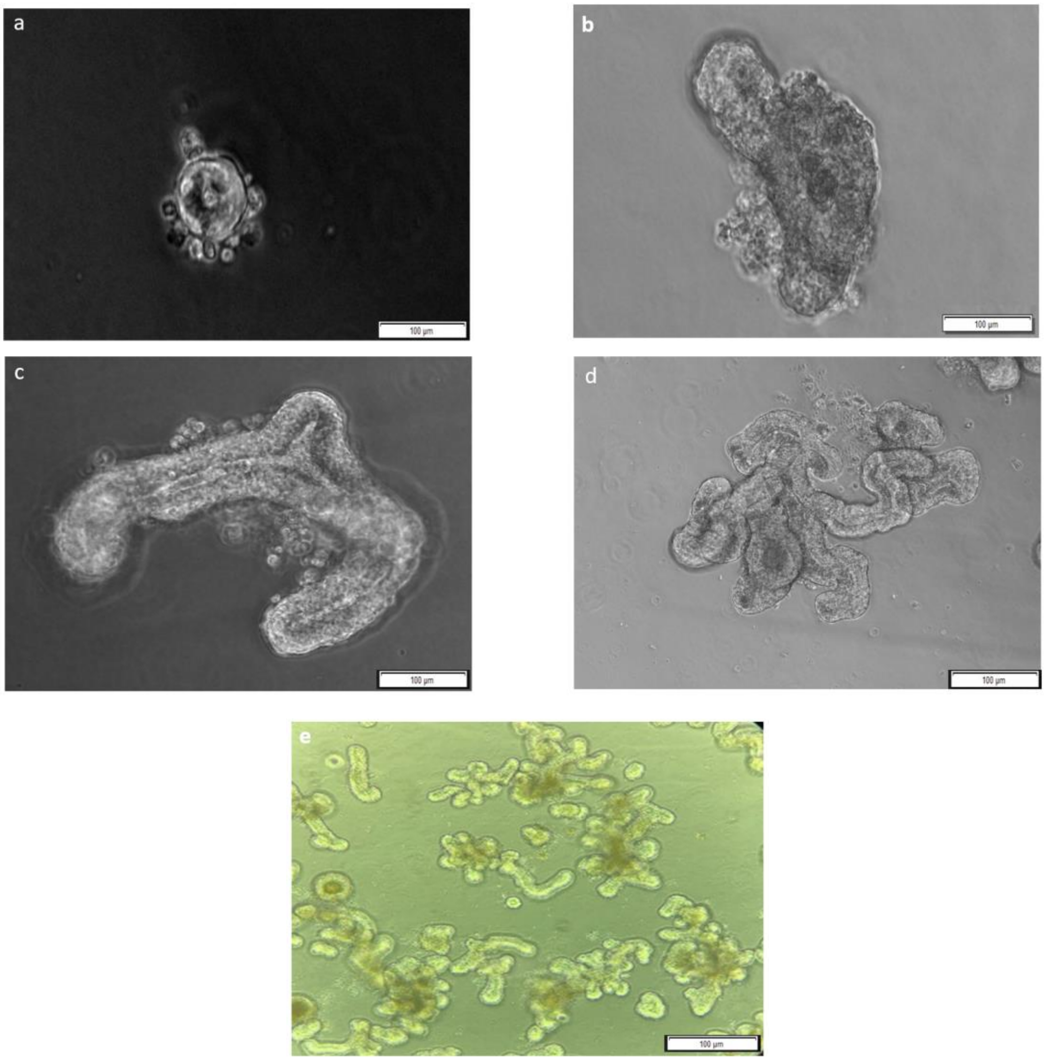


**Figure S1.** Bovine crypts grown in medium 1 forming (a) spheroid-like structures at day 1–2; (b) budding out at day 3; (c) with enlarged branched out structures and lumen, enteroids at day 5; (d) 7 and (e)10.

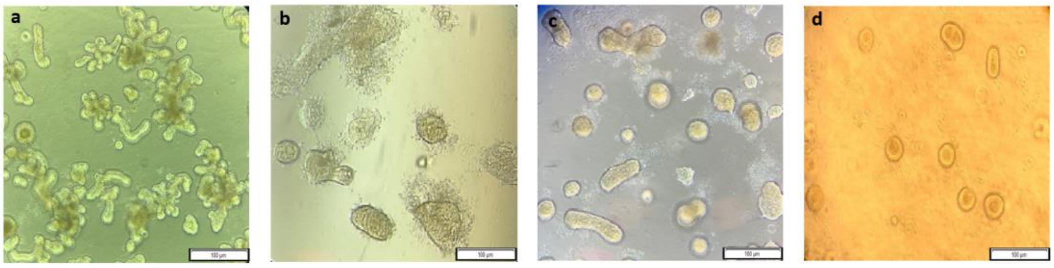


**Figure S2.** Bovine enteroids in four of the tested differentiation media on day 4 (a) Medium 1: enteroids budding out with few dark-centred spheroids; (b) Medium 2: enlarged, rounded, and elongated enteroids with few of them starting to disintegrate; (c) Medium 3: rounded and elongated enteroids surrounded by cellular debris, starting to disintegrate; (d) Medium 4: enteroids appear as round spheroids surrounded by cellular debris.

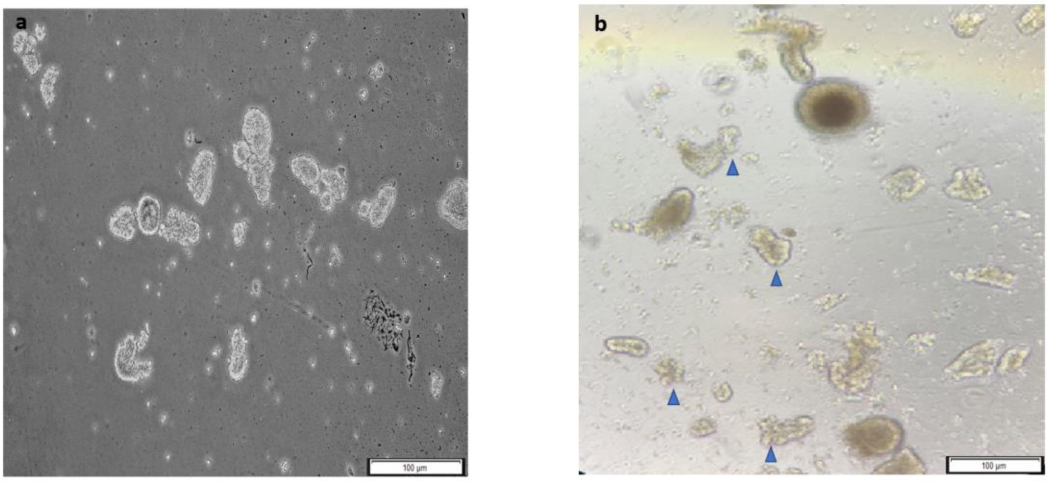


**Figure S3.** (a) Bovine enteroids fragmented by pipetting (setup A) giving finger-like crypts surrounded by some cellular debris. (b) Enteroids further dissociated with Trypl Xpress (setup B) showing more disintegrated crypts with small openings shown with blue arrows surrounded by more cellular debris.

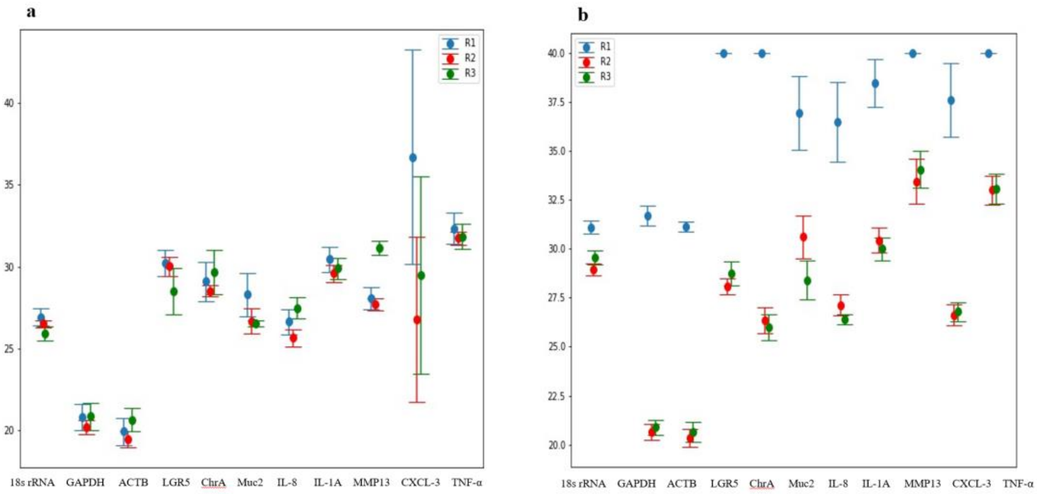


**Figure S4.** Average threshold cycle (Ct) values for eight targeted *Bos taurus* genes of the bovine enteroids either (a) mock-infected or (b) infected with bovine coronavirus at 72 h post inoculation. The dot symbols indicate average mean Ct values while different colours indicate three independent rounds of experiments (R1 = round 1, R2 = round 2, R3 = round 3). The bars indicate standard deviation.
